# Five decades of occupational cancer epidemiology

**DOI:** 10.5271/sjweh.4190

**Published:** 2024-10-01

**Authors:** Michelle C Turner, Kurt Straif, Manolis Kogevinas, Mary K Schubauer-Berigan

**Affiliations:** 1 Barcelona Institute for Global Health (ISGlobal), Barcelona, Spain; 2 Universitat Pompeu Fabra (UPF), Barcelona, Spain; 3 CIBER Epidemiología y Salud Pública (CIBERESP), Madrid, Spain; 4 Boston College, MA, USA; 5 Hospital del Mar Medical Research Institute (IMIM), Barcelona, Spain; 6 International Agency for Research on Cancer (IARC), Lyon, France

**Keywords:** cancer research, cancer type, etiology, occupation, workplace

## Abstract

**Objective:**

In this discussion paper, we provide a narrative review of past and present occupational cancer studies in the journal with a viewpoint towards future occupational cancer research.

**Method:**

We reviewed all references in the journal that mentioned cancer according to relevance to etiology, cancer type, agent type, study design, and study population.

**Results:**

The *Scandinavian Journal of Work, Environment & Health* has published over 300 manuscripts on occupational cancer over the 50 past years. Although studies of cancer represent the primary health outcome in the journal overall, the relative ranking of cancer manuscripts has declined somewhat over time. A large body of evidence from studies of occupation and industry was apparent both in early research and continuing in recent years. There are several examples of the utility of pooled multi-country collaborative studies. Studies also took advantage of available high-quality national population and cancer registers in Nordic countries. There have been notable shifts in focus with regard to the cancer types examined, with increases in publications examining female breast cancer over the decades. The interplay of studies of occupational and environmental cancer has also been apparent.

**Conclusions:**

The journal offers a unique viewpoint to consider the evolution of occupational cancer evidence over time. Studies of occupational cancer have played a central role in global cancer hazard identification efforts. Although much has been gained, there remains a need for renewed global support for occupational cancer research. Concerted efforts will be needed to ensure a future robust evidence-base for occupational and environmental cancer worldwide.

The *Scandinavian Journal of Work, Environment & Health* has published over 300 manuscripts on occupational cancer over the 50 past years. Although studies on cancer represent the primary health outcome in the journal overall, representing 18% of total publications, the relative ranking of cancer manuscripts has declined somewhat over time from being the most common topic between 1975–1984 to the fourth in 2015–2023 behind mental disorders, musculoskeletal disorders, and circulatory diseases (1). Cancer was most often studied in relation to chemical exposures, including organic solvents and pesticides. Studies of occupational cancer were submitted primarily by authors in Nordic countries as well as elsewhere in Europe and North America. Changing trends in occupational health research more broadly were recently described in an analysis of 26 peer-reviewed journals from 1990–2022, with studies of occupational cancer, chemical exposures (lead, asbestos), and organ damage (lung and respiratory) predominant in the 1990s; studies of psychosocial factors, productivity, and biological factors emerged over the 2000s (2).

This paper presents a narrative description of occupational cancer studies published in the journal over the 50 past years with a viewpoint towards future research. We reviewed all references in the journal that mentioned cancer (see hawcproject.iarc.who.int/assessment/918/ for search terms and reference tags). We tagged each reference by relevance to etiology (ie, whether it examined the association between one or more agents and cancer), cancer type, agent type, study design (cohort, case-referent, case report or series, meta-analysis, ecological study), and study population type (industry-based, general population-based, exposure registry-based, hospital or clinic-based). Review articles without a meta-analysis were excluded from tagging but are noted below. Other related publications in this 50-year anniversary series have recently summarized topics in asbestos, solvents, and working hours, and as such they are not covered in detail here (3–5).

## Fifty years of occupational cancer studies

The journal offers a unique viewpoint to consider the evolution of occupational cancer evidence over time. A large body of evidence from studies of occupation and industry was apparent in early studies, shifting to more targeted agent-specific analysis, including complex exposures over time.

## Case reports or series

In some of the earliest publications, Infante et al (6) described three cases of aplastic anemia and acute leukemia with previous occupational or residential chlordane or heptachlor use and five neuroblastoma cases with pre- and post-natal residential exposure to chlordane. Fingerhut et al (7) reviewed seven cases of soft tissue sarcomas among chemical workers with exposure to dioxin-contaminated products, and called for larger, well-designed studies. Difficulties in retrospective exposure assessment through work history records were described as was inadequacy of death certificate information for case ascertainment (7). The International Agency for Research on Cancer (IARC) last convened a Working Group to evaluate chlordane and heptachlor in 2000 and classified them in Group 2B with inadequate evidence in humans, with either no clear findings observed, or multiple co-exposures (8). Classified as a Group 1 agent with sufficient evidence for all cancer sites combined, 2,3,7,8-tetrachlorodibenzo-p-dioxin was the ﬁrst agent classiﬁed initially in Group 1 based on suﬃcient evidence in experimental animals and strong mechanistic data, which was later conﬁrmed by increased cancer incidence in humans (9, 10).

## Industry-based research, including studies of occupations

**Figure 1:**
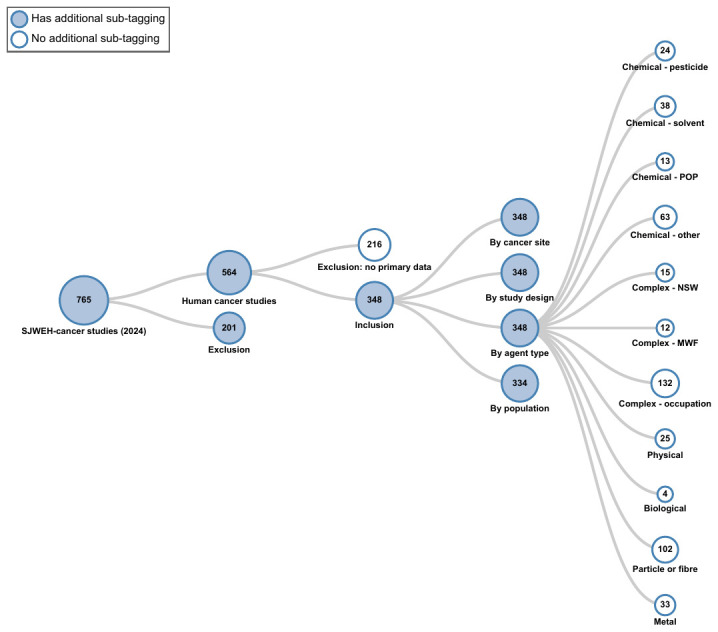
Distribution of etiologic occupational cancer studies by “agent” type. Scandinavian Journal of Work, Environment and Health (2024); Literature tagtree.[MWF=metalworking fluids; NSW=night shift work; POP=persistent organic pollutant.]

Studies of cancer among workers in various occupations and industries have been examined extensively, comprising more than ⅓ of all etiologic cancer studies (figure 1). The number of etiologic cancer studies peaked in the late 1980s and 1990s, largely due to the publication of industry-based cohort studies, many of which were subsequently pooled or meta-analyzed. Despite various methodological limitations (below), historical cohort studies have contributed substantially to the body of evidence and cancer hazard identification efforts (11). Examples of early studies include of railroad workers (12), furniture makers (13), pickling house workers (14), rubber workers (15), mineral wool production workers (16), foundry workers (17), granite workers (18), stone workers (19), shoe workers (20, 21), and thermoelectric plant workers (22). Studies typically examined single or multiple cancer outcomes, comparing cancer incidence or mortality rates among workers by job title overall, or according to task, job area, or exposure status for example, to those of a reference population, often the regional or national population.

Potential limitations have been described (23), including a lack of detailed exposure assessment. Studies were conducted to generate hypotheses and stimulate further investigation into specific exposures in more detail. For example, Acheson et al (13) conducted a retrospective cohort study of furniture makers using employment records. IARC classified work in the furniture and cabinet-making industry in Group 1 in 1987 (24). In 1994, the causal association of work in the furniture and cabinet-making industry and sinonasal and nasopharyngeal cancers were attributed to wood dust (25).

Evaluations of cancer risk among workers in specific occupations or industries typically are more informative in settings with single high-level exposures. In occupational settings with multiple or combined exposures, interpretation of findings and prevention of specific exposures are more complex (26, 27). Potential challenges of disentangling co-exposures were highlighted in a study of railroad workers exposed to herbicides (12). In contrast, studies of granite (18) and stone (19) workers were carried out among those not exposed to other occupational lung carcinogens (PAH, radon). In 1996, crystalline silica dust was classified as a Group 1 lung carcinogen, findings from studies with the least potential for confounding by other occupational lung carcinogens were informative in the evaluation (28). In another example, cancer incidence and mortality were studied among rubber manufacturing workers (15). Occupational exposures in the rubber manufacturing industry were classified as Group 1 with *sufficient* evidence in humans for leukemia, lymphoma, and cancers of the urinary bladder, lung and stomach (10). However, the complexity of exposures in the industry was noted precluding a clear conclusion with a particular chemical. Future studies using detailed exposure data in the industry were suggested.

More recent studies examined cancer risk among seafarers and fishermen (29), offshore workers (30), or firefighters (31), who are exposed to various known or suspected carcinogenic agents and organizational factors. In the case of firefighters, IARC's recent reclassification of this occupation into Group 1, with *sufficient* evidence for mesothelioma and bladder cancer (32), has been followed by extensive prevention efforts, including studies evaluating cancer-related biomarkers of early effect and implementation of the hierarchy of controls to reduce exposure to carcinogens (33–35).

Other limitations may also include lack of information on potential confounders such as personal lifestyle factors. For example, there was concern regarding impacts of cigarette smoking in a study observing elevated rates of lung cancer mortality among foundry workers (17). In contrast, among woodworkers, cigarette smoking maybe less prevalent, and lower mortality rates from lung cancer were observed (13). In a more recent paper of lung cancer risk among coal miners, ore miners and quarrymen from the SYNERGY pooled analysis of population-based case-referent studies, there was detailed personal information on cigarette smoking as well as of work in other at-risk occupations captured (36).

The importance of the reference population has been described (23). For example, in studies of farmers there were both excesses and deficits of cancer compared with the general population (15, 37–39). Limitations of a general population reference group may include a lack of comparability related to personal-level factors, healthy worker bias, or bias in medical diagnostic practices. The impact of the reference population was previously discussed in studies of underground miners (40) or more recently firefighters (31, 32).

## Case-referent studies

Numerous case-referent studies have been published in the journal. Case-referent studies have often been based in registry data (below). One early example is a multi-country case-referent investigation of nasal and sinonasal cancer using data from cancer registers/hospitals in Denmark, Finland, and Sweden, studying several occupational exposures assessed by telephone interview (41, 42). Other examples include of occupation and liver or renal cell cancer in Finland (43, 44) or of occupational solvents and acute myeloid leukemia in four Nordic countries (45). Other case-referent studies have been based in death certificate (46) or burial registry data (47), were hospital-based (48–50), population-based (51–54), or population-based in geographical areas with a predominant industry [such as of agriculture, textiles, or woodworking (47, 55–58)]. There was also a multi-center case-referent study of rare cancers in Europe, including of uveal melanoma (59) and male breast cancer risk (60).

Early efforts to advance occupational exposure assessment in population studies included a multi-cancer, multi-exposure case-referent study of 20 types of cancer designed to identify new occupational cancer risks (61). Exposure assessment was conducted with probing interviews and translation into occupational exposures by trained chemists including confidence that exposure took place, frequency, level, and number of years of exposure. Information regarding 300 common past occupational exposures was captured. Analysis was performed for each cancer site with referents composed of the other included cancer cases in an effort to limit potential recall bias, by using other cancer rather than healthy controls (62, 63). Numerous analyses from the study have been published, including of petroleum-derived liquids (62) and exhaust and combustion products (63).

## Large-scale registry-data

Studies in the journal took advantage of available high-quality national population- and cancer registers in Nordic countries examining cancer incidence and mortality outcomes in large-scale studies (1, 64). In one study, cancer incidence by occupation among 10 million employed persons in Denmark, Finland, Norway, and Sweden was evaluated including of one million incident cancer cases diagnosed during a 20-year period (65). Further, high-quality cancer registries allow examination of trends in disease over time, for example of rates of mesothelioma incidence and predictions of future disease (66, 67). They are also useful to identify cancer cases among exposed populations, produce unbiased case series for case-referent studies, and identify cases of rare cancers (68). In the Italian national mesothelioma registry, Marinaccio et al (69) examined the epidemiology of pericardial and tunica vaginalis testis mesothelioma and associations with occupational asbestos exposure. Another recent study linked farmers’ health insurance and cancer registry data in Taiwan (70). The availability of long-term registry-data also offers the opportunity to examine parental occupational exposures and cancer outcomes among offspring (71–74). Limitations of registry-based studies typically include lack of refined exposure and potential confounder data (75–77). There have been some recent advances in register-based exposure assessment, for instance in using algorithms of working time patterns based on employer electronic records (4, 78).

## Collaborative studies

There are several examples of the utility of pooled multi-country collaborative studies to improve statistical power, especially for rare outcomes, among workers in a range of occupations or industries. These include studies of vinyl chloride workers (79), workers in the production of man-made mineral fibers (80), workers in the reinforced plastics industry with high levels of styrene exposure (81), road pavement and asphalt mixing workers exposed to bitumen fumes (82, 83), and workers in wood-related industries (84). Advances in disease outcome classification have been examined in updated analyses of styrene exposed workers regrouping lymphatic and hematopoietic malignancy outcomes to the latest WHO classification (85). In another example, a pooled analysis of population-based case-referent studies of glyphosate use in North America examined non-Hodgkin lymphoma sub-types in relation to different exposure metrics (86).

## Meta-analyses

A smaller number of meta-analyses have appeared, including several on cancer risks among farmers (87–89) and welders (90, 91). One review compared results among published meta-analyses on night shift work and breast cancer risk to identify research gaps (92). The study reported fairly consistent pooled effect sizes for 'ever versus never' night shift work, with findings for other metrics of exposure inconclusive and pointed to only one meta-analysis (93) that was considered strong in key domains of quality. Future research with more detailed and comparable metrics of night shift work was suggested, as were future high quality meta-analyses that consider in detail quality of individual studies. Accordingly, the carcinogenicity of night shift work was re-evaluated in 2019 and classified as Group 2A, with *limited* evidence for breast as well as prostate and colorectal cancer due to variability in findings and concerns regarding potential bias (94).

## Job-exposure matrices

Studies linking information on job titles to job exposure matrices (JEM) are also prominent in the journal, to assign occupational exposure estimates to study participants using a standardized approach. One study described the utility of a plant- and period-specific JEM based on homogeneous exposure zones for 12 exposures to increase sensitivity and specificity in exposure assessment among wood workers (95). The JEM was based on workplace measurements, expert data, and other historical data and allowed for comparison of several metrics of exposure in internal cohort analyses.

General population JEM, including the expert-based quantitative Finnish JEM (FINJEM), designed to approach quality levels in industry-specific JEM (76), were applied in a census-based study of iron and welding fumes and lung cancer risk (96), in a study of biologically monitored workers for lead to assess potential confounding by other occupational carcinogens (97), and in a hierarchical Bayesian meta-analysis of pancreatic cancer and occupational agents (98). The Nordic Occupational Cancer Study (NOCCA) JEM, an adaptation of the FINJEM by national experts, was used in a large-scale study of solvent exposure and acute myeloid leukemia (45). In one study, a measurement-based quantitative gender-specific JEM for extremely low-frequency magnetic field exposure was applied in an attempt to reduce misclassification from applying a JEM designed for males to females (99).

Most recently, the impact of different JEM dimensions in exposure–response relationships of occupational silica exposure and lung cancer risk was examined in the SYNERGY study (100). Generally similar findings with alternative JEM specifications were observed, though they were optimized in analyses that incorporated prior rating, job, time, and region, with quantitative job-specific estimates being the most prominent dimension.

## Advancing exposure assessment

Previous reviews described the need for advances in exposure assessment beyond job-title based approaches (23, 26, 101), including by integrating quantitative workplace monitoring data, work histories, work practices, and biological monitoring data (12, 102, 103). Detailed retrospective exposure assessment efforts, however, are often resource-intensive (101).

Some studies reported use of workplace measurement data. In one study of aluminum smelter workers, associations of coal-tar pitch volatiles and bladder cancer incidence were based on quantitative estimates of past workplace exposure to total tar (benzene-soluble matter) and benzo-a-pyrene (BaP) based on personal or stationary sampling (104). In a cohort of male metal workers, lifetime individual exposure to welding fume particulates was estimated through questionnaire information and welding-process-specific measurements of fume particulates (105).

One study described a deterministic model for retrospective exposure assessment of phenoxy herbicides, chlorophenols and dioxins, to improve upon expert-based approaches (106). There were a small number of studies of workers biologically monitored for exposure to lead (97, 107, 108) or more recently N,N-dimethylformamide (109). One study characterized metal exposures in lung tissue among deceased smelter workers compared with rural and urban referents (110). An example published elsewhere describes integration of a JEM with serum dioxin concentrations and biokinetic models in an occupational cohort study to estimate dose–response for risk assessment of all-cancer mortality (111).

## Occupation and the environment

The interplay of studies of occupational and environmental cancer has been apparent over previous decades (27, 112–116). Occupational chemicals may spread from the workplace to the general environment (103). One study examined non-Hodgkin lymphoma and soft-tissue cancer incidence among a population previously exposed to chlorophenol, 20-years following the closing of a water intake plant contaminated by a local sawmill (117). In a more recent example among women in North Jutland, Denmark, the main causes of malignant mesothelioma included environmental (distance to industrial source) and domestic (due to living with an asbestos worker) exposure, which may be neglected risk factors (118). Asbestos fibers in artificial clay for toys was also described as a novel exposure source in the journal (119).

Studies of occupational exposures may inform understanding of environmental exposures typically experienced at lower levels. In the case of radon, there were previous studies of lung cancer among zinc-lead miners (120), niobium miners (121), and pyrite miners with low-level exposure to radon daughters (40). Exposure assessment included underground work (40, 120) or categories of cumulated dose of alpha radiation based on underground working time and mean underground measurement levels (121). There were also studies of residential radon by residency type (as an indicator of radon exposure) in a rural area (122), and with estimated or measured exposure levels to radon daughters in homes (123, 124). Potential interactions of smoking, passive smoking, and radon were examined (120, 123, 124). Occupational exposure to radon and its decay products were classified in Group 1 with *sufficient* evidence for lung cancer in 1988 (125). In 2001, the consistency of estimates from studies of underground miners and the growing body of literature on residential exposure was highlighted (126). In 2006, Darby et al (127) reported results of a pooled analysis of residential radon and lung cancer from 13 case-referent studies in Europe with information on individual smoking histories and long-term residential radon gas measurements. A recent study of childhood leukemia was based on predicted indoor radon concentrations, however future studies using measured rather than modeled data were suggested (128).

Studies of residential exposures can also vice versa, inform occupational exposures. For passive smoking, not fully conclusive information on health effects of workplace exposure was described (129). Later in the 2002 IARC evaluation of involuntary smoking (classified in Group 1), published meta-analyses reported a 12–19% increase in lung cancer risk among never smokers exposed at the workplace, though the smaller evidence base of studies of workplace compared with residential exposure was noted (130).

## The changing landscape of occupational cancer research

**Figure 2:**
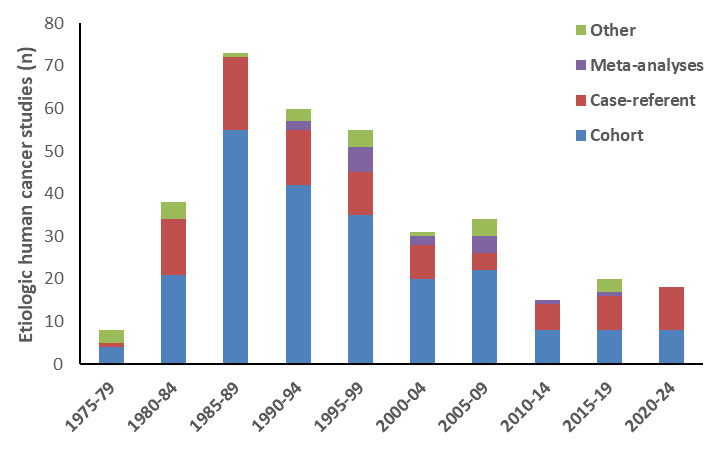
Frequency of etiologic occupational epidemiology studies published in the Scandinavian Journal of Work, Environment and Health, by year and study design.

**Figure 3:**
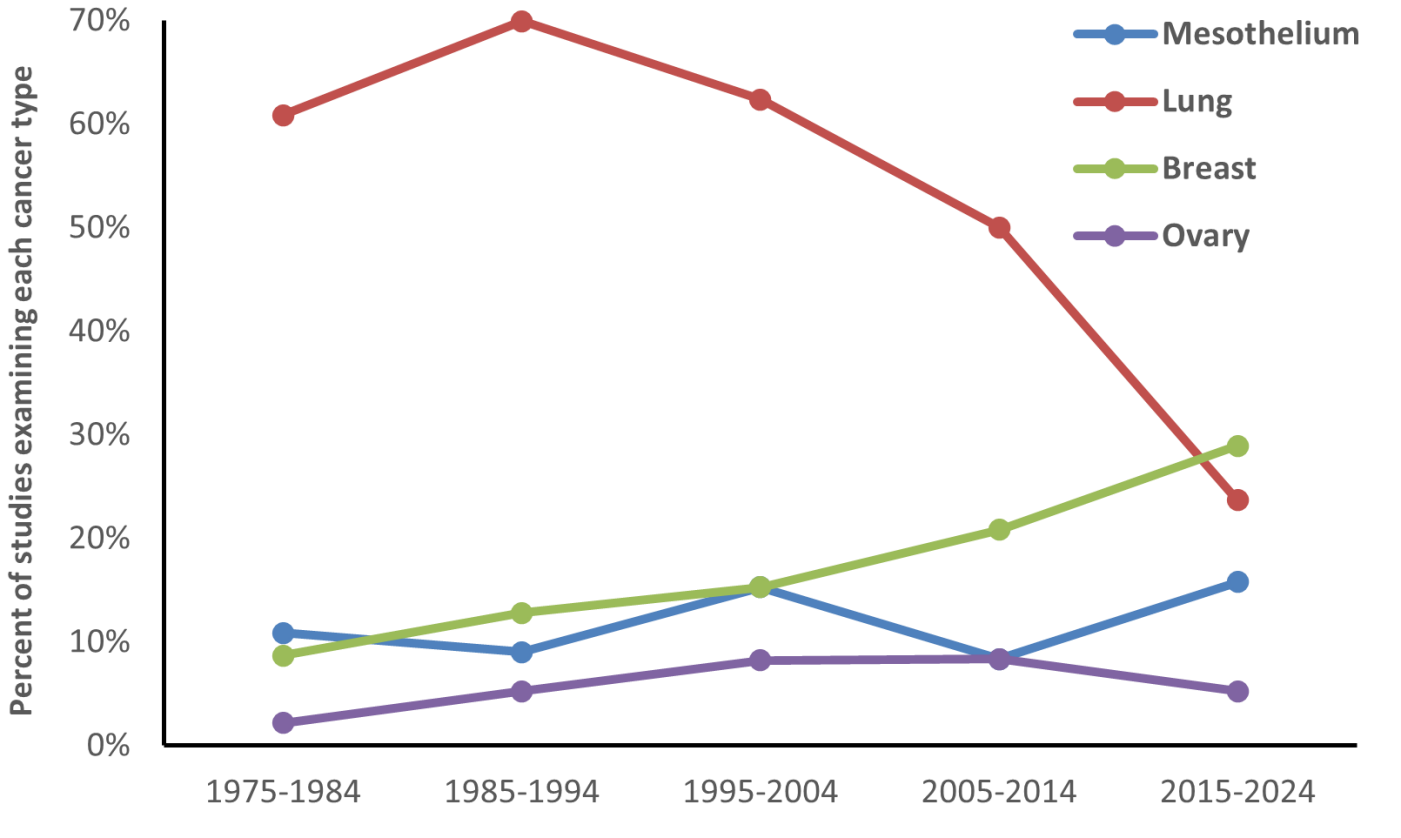
Percentage of etiologic occupational studies examining cancers of mesothelium, lung, breast, and ovary in Scandinavian Journal of Work, Environment and Health over the decades (includes multiple cancer outcomes in cohort studies).

The evolution of study designs across the 50-year history of the journal can be seen in figure 2. Since the early 1980s, the number of case-referent studies has been fairly stable, with large, pooled population-based studies published since the early 2010s. Most meta-analyses on cancer were published between 1990 and 2009. More stringent editorial criteria resulted in fewer meta-analyses published more recently. There have also been notable shifts in focus with regard to the cancer types examined (figure 3). Interest in mesothelioma has remained steady, while studies examining lung cancer declined. Publications describing breast and ovarian cancer increased, reflecting inclusion of women in cohort studies and detailed examination of these cancers in case-referent studies.

There have also been shifts in topics with the most recent studies encompassing a broader range of topics and methods. Although studies of chemicals, particles, and fibers remained, recent etiologic studies also covered shift work (4), noise (131), biological agents (132, 133), heat (134), complex mixtures (eg, welding and early cancer-related biomarkers) (135) or the psychosocial work environment. In a study of job demands in the SYNERGY study, there were stronger associations for lung cancer with higher physical rather than psychosocial demands, likely due to capturing undetermined effects of occupational lung carcinogens (136). There were also recent studies of labor force participation and return-to-work among cancer patients. One study examined predictors of employment among cancer survivors, highlighting the importance of motivation and skepticism towards returning to work (137). Other studies highlighted the need for improved support from healthcare professionals, supervisors and colleagues (138–140).

## Future research on occupational cancer

Studies of occupational cancer have played a central role in global cancer hazard identification efforts including identification of 61 Group 1 occupational carcinogens (including exposure circumstances) having one or more sites with *sufficient* evidence of carcinogenicity, nearly half of the 129 Group 1 agents (11). This includes findings from recent evaluations that identified two additional occupationally relevant Group 1 agents including occupational exposure as a firefighter [*sufficient* evidence for mesothelioma and bladder cancer and *limited* evidence for several other cancer types (32)] and acrylonitrile [*sufficient* evidence for lung cancer and *limited* evidence for bladder cancer (141)]. Chemicals and chemical mixtures, radiation and radionuclides, and airborne particles represented the most frequent occupational carcinogens, and lung cancer the most frequent cancer type (11).

Although much has been gained in 50 years of occupational cancer studies, there remains the need for renewed global support for occupational cancer research (142–144). The declining trend in published cancer research observed in the journal may at least partially reflect earlier calls for renewed funding for cancer studies of this type (144). There remain many agents with *limited* human cancer evidence and an even larger number with *sufficient* evidence of carcinogenicity in experimental animals and potential occupational exposure, and global efforts to highlight research gaps to resolve classification uncertainties are needed (143, 144). There are large numbers of workers exposed to Group 1 agents (145–147), as well as new chemical hazards and changing workplace tasks over time (148).

Priorities for evaluation by the *IARC Monographs* may influence short- and longer-term research agendas; one example is the large number of studies of night shift work in the journal in close proximity to the *Monographs* evaluation (4). In a recent effort to recommend priority agents for future evaluation by *IARC Monographs* for 2025–2029, the accrual of pesticides as high-priority agents for evaluation was described, as was the need for systematic appraisal of all Group 1 agents to identify new cancer sites with *sufficient* or *limited* evidence (149). The importance of maintaining and strengthening global cancer hazard identification efforts, free from corporate and political interests, was highlighted some twenty years ago (150, 151), and remains today (152). The recent transition of the journal to full open access is also an important development, facilitating access to occupational cancer studies globally (153).

There remains a need for new studies to identify emerging risks, including an emphasis on potential impacts of climate change on occupational cancer risk. Climate change will lead to changes in frequency or intensity of a range of hazardous exposures and tasks (154). The future of occupational cancer research will take place amidst a changing workplace and green transition (155). The ongoing transformation and reorientation of global economies towards sustainability, includes the elimination and transformation of jobs and changes in exposure to known and unknown hazardous agents, and has yet to be fully understood (156, 157). One example includes electronic waste work, predominant in low- and middle-income countries, often unregulated and informal work, where the infrastructure is poor to support the types of studies that have been influential in reaching *sufficient* evidence (149).

Advances in exposure science and exposome methods to characterize the occupational (and non-occupational) environment will contribute to the literature (158). However, gains in hazard identification will also result from continued investment in the types of historical cohort studies outlined above, which have already greatly informed the field. Efforts to support continued updating of follow-up, refining exposure assessment, and to consider potential confounders either directly or indirectly are needed, as are efforts to support new cohorts on novel exposures, and studies with up-to-date outcome classification (85, 86). Industry-based studies, even if decades old, are frequently considered highly informative, and have been important in moving human cancer evidence from inadequate to limited or even sufficient (figure 1.4 in 159).

There is need for further studies of women and vulnerable populations. Although studies including female breast or reproductive cancers have typically not formed a large proportion of studies in the journal, the substantial increase in studies of female breast cancer in more recent decades has been observed (figure 3). Other potentially vulnerable populations, with greater exposure to occupational carcinogens include workers in smaller companies and migrants (146).

There is need to study effective workplace interventions for cancer prevention. Few studies of primary or secondary cancer prevention were published in the journal. One previous review described health promotion trials for cancer risk factors at the workplace (160). Another publication discussed recommendations for prevention of adverse effects of night work for breast cancer (161). One study evaluated cost-effectiveness of low-dose computed tomography screening among asbestos-exposed workers (162). In other work, occupational health researchers were encouraged to follow the field of lung cancer screening and advance understanding regarding which workers will benefit (163). Concerted efforts among the occupational cancer research and broader interdisciplinary community are needed to ensure a robust future evidence-base for occupational and environmental cancer worldwide.

## Disclaimer

Where authors are identified as personnel of the IARC/WHO, the authors alone are responsible for the views expressed in this article and they do not necessarily represent the decisions, policy or views of the IARC/WHO.
